# Love Thy Neighbour: Automatic Animal Behavioural Classification of Acceleration Data Using the K-Nearest Neighbour Algorithm

**DOI:** 10.1371/journal.pone.0088609

**Published:** 2014-02-21

**Authors:** Owen R. Bidder, Hamish A. Campbell, Agustina Gómez-Laich, Patricia Urgé, James Walker, Yuzhi Cai, Lianli Gao, Flavio Quintana, Rory P. Wilson

**Affiliations:** 1 College of Science, Swansea University, Swansea, Wales; 2 School of Biological Sciences, University of Queensland Brisbane, Queensland, Australia; 3 Centro Nacional Patagónico - Consejo Nacional de Investigaciones Cientificas y Técnias, Puerto Madryn, Chubut, Argentina; 4 College of Engineering, Swansea University, Swansea, Wales; 5 School of Management, Swansea University, Swansea, Wales; 6 School of Information Technology and Electrical Engineering, The University of Queensland Brisbane, Queensland, Australia; 7 Wildlife Conservation Society, Ciudad de Buenos Aires, Argentina; Tulane University Medical School, United States of America

## Abstract

Researchers hoping to elucidate the behaviour of species that aren’t readily observed are able to do so using biotelemetry methods. Accelerometers in particular are proving particularly effective and have been used on terrestrial, aquatic and volant species with success. In the past, behavioural modes were detected in accelerometer data through manual inspection, but with developments in technology, modern accelerometers now record at frequencies that make this impractical. In light of this, some researchers have suggested the use of various machine learning approaches as a means to classify accelerometer data automatically. We feel uptake of this approach by the scientific community is inhibited for two reasons; 1) Most machine learning algorithms require selection of summary statistics which obscure the decision mechanisms by which classifications are arrived, and 2) they are difficult to implement without appreciable computational skill. We present a method which allows researchers to classify accelerometer data into behavioural classes automatically using a primitive machine learning algorithm, k-nearest neighbour (KNN). Raw acceleration data may be used in KNN without selection of summary statistics, and it is easily implemented using the freeware program R. The method is evaluated by detecting 5 behavioural modes in 8 species, with examples of quadrupedal, bipedal and volant species. Accuracy and Precision were found to be comparable with other, more complex methods. In order to assist in the application of this method, the script required to run KNN analysis in R is provided. We envisage that the KNN method may be coupled with methods for investigating animal position, such as GPS telemetry or dead-reckoning, in order to implement an integrated approach to movement ecology research.

## Introduction

The use of animal attached sensors for monitoring animal movements and behaviour is now common practice (see [1] for review). In particular, accelerometers attached to animals allow the measurement of animal energy expenditure [2], [3], [4], [5], travel speed [6], [7] and behaviour [8], [9] in environments which preclude direct observation, thus saving time and field effort (for details see [10]).

With the development of a movement ecology paradigm seeking to integrate information of animal location, behaviour, energy expenditure and environmental information [11], animal-attached accelerometers show great promise as part of the movement ecology ‘toolbox’, because they can be used to study both the behaviour and energy of free-living animals [10], [12], [13]. Indeed, an increasing number of studies are making use of accelerometers to quantify animal behaviour [14], [15], [16], [17]. Most of these studies identify behaviour following the principles set out in Shepard *et al.* [18]. This method requires that researchers go through the data manually and interpret the signals according to changes in body posture and body motion, both of which are discernable using accelerometers [15], [18]. Body posture can be detected as ‘static’ acceleration, and relates to the orientation of the accelerometer with respect to gravity. Body motion is detected as ‘dynamic’ acceleration when the inertia produced by animal movement registers characteristic signals on the device [18]. However, modern accelerometer-equipped data loggers are now able to record at rates as high as 300 Hz [19], so manual identification of behavioural patterns in accelerometer data using this approach is arduous and, with increases in the use and capacity of the technology, is set to become more so.

Some formalised procedures may help with this issue. For example, a simple method of automatic classification involves labelling data into behaviours by a sequence of rules (i.e. do data values exceed a given value), called thresholding: Moreau *et al.* [20] used threshold values to delineate whether goats *(Capra aegagrus hircus)* were grazing or browsing (head-up or -down state) while a similar approach was adopted by Lagarde *et al.* [21] in a study of the Greek tortoise (*Testudo graeca*) activity. In the latter study, a series of decision rules were designed through visual observation of the subject animals to discriminate between five behaviours, with high accordance between estimated and observed behaviours [21]. However, the effectiveness of threshold methods are limited by the need for accurate selection of threshold values in the first instance, something that can only be achieved through visual observation and familiarity with the subject species. This issue was addressed by Sakamoto *et al.* [*22*] through the use of K-means clustering, which attempts to discover behavioural modes in the data automatically through unsupervised machine learning without ground-truthing. Unfortunately not all behaviours were discernible by the method, and it was limited to input from a single acceleration axis [22].

Another approach that has shown promise is the use of Machine Learning Algorithms (MLAs), specifically Support Vector Machine (SVM) algorithms. SVMs are a form of binary classifier, which differentiate between behavioural modes by representing data as points in space based on summary statistics derived from training data (*i.e.* the data collected under observation which is used to provide the machine learning algorithm with an example of data pertaining to a given behaviour) [13]. A hyperplane (or division) is drawn at the maximum distance (usually Euclidean distance) between each training class, and new data are classified according to which side of the hyperplane they fall. Because SVMs are binary classifiers, *i.e.* they can only differentiate between two classes at a time, the problem must be split into multiple binary classifications when there are more than two behaviours, i.e. behaviour *A* or all others, behaviour *B* or all others, *etc.* [13].

To our knowledge, the first study to illustrate the utility of SVMs in the classification of accelerometer data into animal behavioural states was Martiskainen *et al.* [23]. In this study, SVMs were applied to accelerometer data obtained from dairy cows in order to distinguish between eight routine behaviours. However, some behaviours returned poor precision (for definition see Methods) in some classifications, due, in part, to similarity in movement patterns between behaviours [23]. Gao *et al.* [24] more recently evaluated SVMs as a means to classify accelerometer data. However, this method involves the use of a web-based program to conduct the analysis, which restricts the input sample rate to 1 Hz. Commonly, this is considered below a useful required sample rate [24], [25], as the sample rate is required to be twice that of the fastest expected movement [26].

Nathan *et al.* [13] evaluated 5 machine learning algorithms (Artificial Neural Networks, Classification and Regression Trees, Linear Discriminant Analysis, Random Forest and Support Vector Machine) for use in classifying acceleration derived from Griffon vultures. Whilst all of the methods tested performed quite well (80–90% accuracy), we would argue that their adoption by the scientific community will be problematic because they are conceptually complex and their efficacy relies on the proper selection of summary statistics. One criticism that is often levelled at machine learning algorithms is that they are ‘black box’ methods that are difficult for biologists to implement or appreciate how classifications are derived.

In light of this, we see a need for a method for automatic identification of behavioural modes that is accessible and straightforward conceptually. The K – Nearest Neighbour (KNN) algorithm [27], [28] is such a method, by which new data are classified according to the classifications of the *k* nearest data points from a training set [29]. This training set can be derived from ground-truthed data obtained under visual observation (e.g. [30]). KNN is a form of primitive machine learning, and can be used to classify raw acceleration data according to its position in a 3 d feature space and, compared with other MLAs, it is intuitive and computationally simple [31]. The KNN is an established method in data classification and has been used in numerous fields, such as microbiology [32], security [33], forestry [34] and hydrology [35].

The purpose of the present paper is to introduce KNN as an easy to use and conceptually simple method for identifying animal behavioural modes in raw tri-axial acceleration data. The method detailed here requires no specialist coding experience or selection of summary statistics to implement, and can handle high sample rate data (up to 40 Hz are tested here). KNN analysis can be carried out with the freeware program R, with the script provided (see File S1). In order to evaluate the utility of the KNN method, we used the algorithm in R to discern between five common behaviours of 8 species; Human (*Homo sapiens*), Badger (*Meles meles*), Cormorant (*Phalacrocorax atriceps*), Cheetah (*Acinonyx jubatus*), Camels (*Camelus dromedarius*), Dingo (*Canus lupus dingo*), Kangaroo (*Macropus rufus*) and Wombat (*Lasiorhinus latifrons*).

## Methods

### K-Nearest Neighbour Algorithm

**Figure 1:**
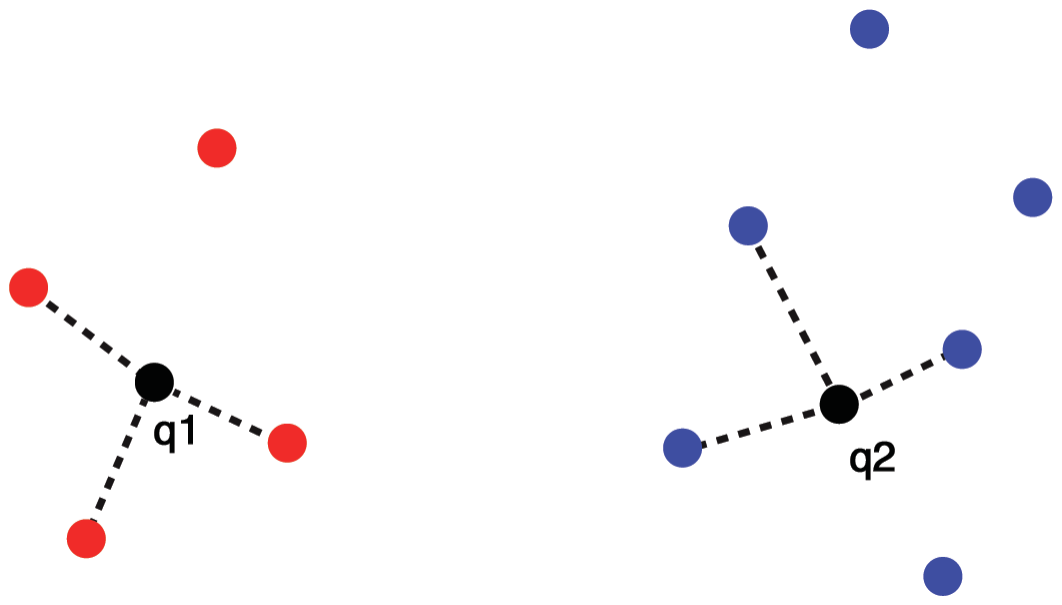
Simple example illustrating KNN analysis when *k* = 3. Here the new data points are classed according to a majority vote of their *k* nearest neighbours, so q1 is classed as red and q2 as blue. Two variables are used in this example, although the same approach may be used with *n* dimensional data such as tri-axial accelerometer data.

The concept behind KNN is intuitive; new data points are classed according to the classes of the points which are closest to them in the training data. KNN is a primitive form of machine learning that is often referred to as ‘lazy learning’ because induction occurs during run time [36]. Figure 1 illustrates a simple example classification. In this example, *k* is set to 3 so the three nearest training data points to new points q_1_ and q_2_ determine the classes of these points by majority vote. In this example, q1 is classed along with the red points and q2 along with the blues. Thus the KNN method may be separated into two stages; first, for attribute or dimension *r* (the variable, in our case acceleration in *g*) the Euclidean distance, *d,* between new data point *x_i_* and training data point *x_j_* is calculated by the formula given in Mitchel [37];
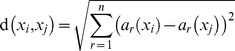
(1)


The algorithm then selects the *k* number of values with the least Euclidean distance. Note that Euclidean distance is used because it is the convention with KNN, although other distance metrics may be used [38]. If these *k* nearest values (or *k* nearest neighbours) are of two classes *a* and *b*, class *a* will be selected when the number of points belonging to class *a* outnumber those of class *b*, or *n_a_>n_b_.* The KNN algorithm is present in the R package *class*, and also provides the output value *prob*, which is the proportion of *k* nearest values in the training set that belonged to the winning class.
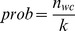
(2)where *n_wc_* denotes the number of points in the winning class. In order to improve accuracy, a threshold filter can then be applied to the *prob* values to produce a minimum majority threshold. Classifications made by the KNN that do not surpass this threshold are discarded. In the field of machine learning, algorithms are often evaluated through the construction of a confusion matrix [39], a table that visually represents correct and incorrect classifications. Through construction of a confusion matrix it is possible to count how many *true positive, false positive, true negative* and *false negative* classifications are made. For use in the confusion matrix, classifications that surpass the threshold, and are verified as correct are taken as *true positive (TP)*. Classifications that surpass the threshold but are verified as incorrect are taken as *false positive (FP)*. Classifications that were verified incorrect and did not meet the threshold are taken as *true negative (TN)*, and those correct classifications that do not meet the threshold are *false negative (FN)*. These values are then used in order to calculate the performance metrics, Accuracy, Precision and Recall (see Evaluation Procedure).

### Evaluation Data Sources

In order to evaluate KNN as a method for classifying tri-axial accelerometer data according to behavioural modes, data were collated from various sources (Table 1). A detailed account of tagging procedures of cheetah, dingo, kangaroo, wombat, badger and cormorant can be found in the source studies, given in Table 1. These studies was carried out under a University of Queensland Animal Ethics permit (SBS/300/12) and badger monitoring conducted under Natural England Badger Licence No. 20112793 held by the RSPCA, UK. The Camel deployment protocol was evaluated and approved by Lokhit Pashu-Palak Sansthan, India. Cormorant fieldwork at Punta Leon was conducted under permit from Organismo Provincial de Turismo, Argentina. The experimental protocol for the human subject was approved by the ethics committee of Swansea University, and the participant gave written informed consent.

**Table 1:** Descriptions of species used, behaviours performed, and sources of data.

Species	Source	Sample Rate	Behaviours
			Sit
Cheetah			Stand
Dingo	Campbell *et al.* 2013	20 Hz	Rest
Kangaroo			Run
Wombat			Walk
			Forage
Badger	Gao *et al.* 2013	20 Hz	Rest
			Run
			Walk
			Climb
			Rest
Camel	Swansea University	40 Hz	Stand
			Walk
			Graze
			Browse
			Stand
Human	Swansea University	20 Hz	Lying
			Walk
			Run
			Crawl
			Dive Ascent
Cormorant	Gomez-Laich*et al.* 2008	20 Hz	Dive Bottom
			Dive Descent
			Flying
			Walk

The tagging procedure used to obtain the data from the camel and human are currently unpublished, and so is presented here. A Dromedary Camel (*Camelus dromedaries*) of the “Mewari” breed [40] was equipped with a Daily Diary data logger [10] at Lokhit Pashu-Palak Sansthan centre, in Rajasthan, India. The device was set to record at a sampling frequency of 40 Hz, at 12-bit resolution. The device was attached to a collar that hung below the neck, in order for it to become inclined if the animal raised or lowered its head. Whilst being observed, the camel was allowed to roam freely within a field. Behaviour was recorded for this time period, and five behaviours selected for use in this study according to availability of sufficient data to produce training and testing files. These behaviours were ‘Rest’ (sternal recumbency), ‘Walk’ (locomotion on all four limbs), ‘Idle’ (motionless on all four limbs), ‘Browse’ (feeding on trees), and ‘Graze’ (feed from the ground). Other behaviours were performed during observation periods, but not for sufficient time or occasions to allow for inclusion in the analysis.

A human participant was equipped with a X2 mini accelerometer (Gulf Coast Data Concepts, USA) which was held between the shoulder blades using a Silastic® harness (Dow Corning Corporation, USA). The participant was then instructed to perform the behaviours in turn, for a duration of 60 s each. The behaviours were ‘Stand’ (stood still and upright), ‘Lying’ (sternal recumbency), ‘Run’ (locomotory gait with ‘suspended phase’, in which neither foot touches the ground), ‘Walk’ (locomotory gait without a ‘suspended phase’) and ‘Crawl’ (locomotion on hands and knees). This sequence was repeated on two occasions in order to obtain data for training and testing sets.

### Evaluation Procedure

Data for five behaviours (see Table 1) from each species were obtained on two separate occasions, one each for training and testing the KNN algorithm. Both training and testing segments contained 10 s each for every behaviour, equivalent to 1000 and 2000 data points at 20 and 40 Hz respectively. The raw data for all three axis of acceleration pertaining to all 5 behaviours were combined in a single file and labelled for use as training data for the algorithm, and separate instances of the same behaviours were combined for testing data and behaviour labels stored for later verification of the results. Manual observation (human) or video footage (captive animals) was used to find when each of the behaviours occurred, apart from the cormorant, for which behaviours were identified manually [15], [18], a process made particularly robust since it used other sensor data, such as hydrostatic pressure, to help discrimination.

Results from the KNN analysis were then compared to the actual behavioural classification of the data in order to obtain overall accuracy. Following this, a minimum majority threshold was applied to the results. A minimum majority threshold represents a minimum value for the output *prob*, which if not reached, results in the KNN classification being discarded. Thresholds of 0.9, 0.8, 0.7, 0.6 and 0.5 were applied and accuracy, precision and recall were calculated [41]. ‘Accuracy’ was defined as a measure of the overall proportion of correctly assigned data points, and was calculated as;
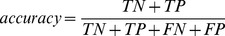
(3)


‘Precision’ was defined as the proportion of positive classifications that were correct, and was calculated as;
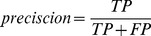
(4)


‘Recall’ was the proportion of data pertaining to behavioural modes that were classified correctly as positive, and was calculated as;
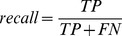
(5)


## Results

All 5 behaviours were detected using the KNN method trialled on all species, except for the kangaroo which was not tested for the ‘Sit’ behaviour because its incidence was not discernible from the video footage. The minimum majority threshold that yielded the highest Accuracy, Precision and Recall differed between species (Table 2) (a detailed breakdown of the Accuracy, Precision and Recall scores for each species is given in Table S1). Generally, 0.7 was the threshold that produced the greatest mean accuracy across all 8 species (mean = 0.781±0.0948). The highest mean precision was observed when the minimum majority threshold was set to 0.9 (mean = 0.902±0.145), and the highest Recall at 0.5 (mean = 0.984±0.012). In all species, increasing the minimum majority threshold resulted in a decrease in the proportion of the data that was classified (Table 3). Increasing the minimum majority threshold improved accuracy for the badger and kangaroo only and there was a negative correlation between threshold level and accuracy for camel and wombat (Table 3). Precision was improved for all species except in the case of the wombat when minimum majority thresholds were increased. There was a negative correlation between minimum majority threshold and Recall in all species (Table 3).

**Table 2:** Highest values of performance measures for KNN on each species and the threshold values used to obtain them.

	Highest Score					
Species	Accuracy	Threshold	Precision	Threshold	Recall	Threshold
Badger	0.71	0.9	0.95	0.9	0.99	0.5
Camel	0.82	0.6	0.90	0.9	0.99	0.5
Cormorant	0.77	0.7	0.87	0.9	0.99	0.5
Cheetah	0.77	0.7	0.90	0.9	0.97	0.5
Dingo	0.83	0.6	0.97	0.9	0.98	0.5
Kangaroo	0.91	0.9	0.97	0.9	1.00	0.5
Wombat	0.76	0.5	0.77	0.9	0.97	0.5
Human	0.95	0.5	0.98	0.9	1.00	0.5

**Table 3:** Results of Spearman’s Rank Correlation between Minimum Majority Threshold value and the resulting performance measures.

	Proportion Classed		Accuracy		Precision		Recall	
Species	r	P-value	r	P-value	r	P-value	r	P-value
Badger	−0.974	0.005	0.963	0.009	0.981	0.003	−0.977	0.004
Camel	−0.999	<0.0001	−0.947	0.015	0.998	<0.0001	−0.996	<0.0001
Cormorant	−0.974	0.005	−0.767	0.13	0.996	<0.0001	−0.947	0.015
Cheetah	−0.999	<0.0001	−0.841	0.74	0.995	<0.0001	−0.998	<0.0001
Dingo	−0.998	<0.0001	−0.859	0.62	0.99	0.001	−0.994	0.001
Kangaroo	−0.996	<0.0001	0.979	0.004	0.994	0.001	−0.948	0.014
Wombat	−0.999	<0.0001	−0.998	<0.0001	−0.43	0.946	−0.999	<0.0001
Human	−0.985	0.002	−0.851	0.067	0.995	<0.0001	−0.972	0.006

## Discussion

The purpose of this study was to illustrate that the KNN method could be used to identify automatically the behavioural modes of animals equipped with accelerometers recording at high sample rates, and that this approach is applicable for large, complex datasets. Our results show that animal behavioural modes can indeed be successfully identified automatically using the KNN method and that, with a mean Accuracy of 78%, they are comparable to results gained using more complex automated methods [13], [24].

**Figure 2:**
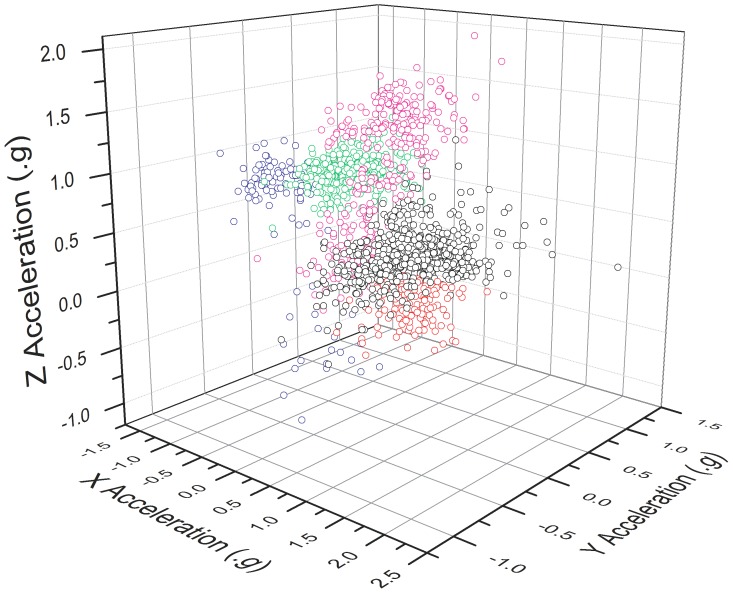
3D Scatterplot showing raw tri-axial acceleration data for an Imperial cormorant (*Phalacrocorax atriceps*), data points are labelled by colour according to their behavioural classification. Red – Ascent Phase of Dive, Green – Bottom Phase of Dive, Blue – Descent Phase of Dive, Purple – Flight, Black – Walking.

Despite the efficacy of machine learning algorithms for classifying animal behaviour automatically [13], [24], [30], we argue that the nature of ‘black box’ algorithms, including the selection of numerous summary statistics, fogs the relationship between animal movement and behavioural classification [42], [43]. Other methods such as Sparse Representation presented by Liu *et al.* [44] alleviate the need for selecting summary statistics and indeed are purported to be more accurate than KNN when used to classify human activities. However, Liu *et al.* [44] were not explicit whether they implement a thresholding filter for KNN as introduced in the present study, but they report a much lower accuracy than that found for humans here (Table 2). It is also relevant that whilst Sparse Representation does not require manual selection of summary statistics by the researcher, the method selects features for analysis automatically and the relationship between data and their classifications are no less opaque than for other ‘black box’ algorithms. One of the strengths of the KNN method is its conceptual simplicity. Figure 2 shows how, if raw acceleration values for each axis are plotted as a 3D scatter plot, the relationship between a data point’s classification and its position in the 3D feature space becomes evident. Understanding this link between animal behaviours and the signals they produce is important for interpretation, diagnostics, and elucidation of behaviours which might have been previously unknown.

Successful implementation of the KNN method requires high quality training data. This training data must be manually classified in the first instance, and it must include sufficient examples of all behavioural modes expected during device deployment. As the KNN makes classifications based upon the position of the data within the 3D feature space (Figure 2), these areas must be sufficiently populated in the training data in order to ensure that accurate classifications are made. It is anticipated that complex behaviours, which include multiple postures or body orientations, may require more training examples in order to establish a sufficient density of data within the 3D feature space. Additionally, it is possible that undefined behaviours may be incorrectly classified, as KNN lacks the capacity to recognise novel behavioural modes (c.f. [23], [24], [45]). In addition, the requirement for a period of observation to obtain training data may be problematic if few captive specimens are available *e.g.* the Ethopian wolf, *Canis simensis* [46]. In instances such as this, it may be possible to use similar species as surrogates in a manner similar to Campbell *et al.* [30]. Despite these requirements, the KNN methods proposed in the present study has the potential to perform behavioural classification far faster and more objectively than manual inspection of acceleration data [15], [18].

Not all recent studies on automatic classification of acceleration data make use of Accuracy as a sole measure of performance (e.g. [23]), which makes comparisons between studies problematic. Selecting performance metrics is challenging because varying the minimum majority threshold has different effects for each species and metric (Table 2). The optimum metric for evaluation of classification algorithms is dependent on the questions being asked and the importance of the various parameters are highly study specific. For comprehensive consideration of this, the metrics are evaluated in Powers [41]. Briefly, Accuracy takes into consideration all classification outcomes; including the true negative rate (*i. e.* data that are erroneously classified by the KNN is discarded because they do not meet the minimum majority threshold). It is a general measure of performance for the classification method and is a simple metric by which different algorithms may be compared. However, when thresholding is used Accuracy values can be high with few usable classifications made. This may occur if many incorrect classifications (produced by the KNN) are correctly discounted by the thresholding filter, resulting in few True Positive classifications but many True Negatives. As a result, this may not be the most effective performance measure for machine learning algorithms in the context of behavioural classification. Alternatively, Precision should be used, as it represents the proportion of positive classifications that were true. We argue that this metric is most appropriate because biological inferences are derived from the positive results (estimations of when behaviours occur) more often than negative ones. However, it is recommended that all three performance metrics are reported when novel classification methods are presented, in the interest of transparency and so that researchers can select methods based on the requirements of their studies.

One might assume that applying a minimum majority threshold of 0.9 would yield the best results because this is the threshold that consistently produced the highest Precision for all species (Table 2). This assumption must also be tempered with the consideration that applying a higher threshold results in more classifications made by the KNN being discarded (Table 3). For example, increasing the threshold for the wombat classifications from 0.5 to 0.9 produced only a 0.3% increase in Precision, yet 37.7% less of the data set met the threshold to be classified (Table 2). This leads us to conclude that threshold levels should be selected according to species after a preliminary period of trial and error. We advocate collecting some additional data during the period of observed ground-truthing (required in any case to produce the training set) for this purpose prior to running KNN on data derived from wild individuals.

The KNN classification of the badger achieved the lowest Accuracy score of all animals tested in the current study. During visual observation, it was noted that the position of the collar on which the accelerometer was mounted altered position. It is possible that this movement may have produced appreciable noise in the accelerometer data through altering the orientation of the device when behaviours were performed. This change in device orientation would have produced a difference in static acceleration [18] recorded. The total acceleration experienced by the accelerometer may be conveniently described as a product of both static acceleration, *i.e.* acceleration due to gravity, and dynamic acceleration, *i.e.* acceleration derived from the animal’s movements [12]. Thus, it is possible for the animal to perform the same movements or behaviour, but record different total acceleration values if the device orientation is not constant [10], [18]. This difference in raw values would explain the low accordance between training and testing data sets during KNN analysis for the badger. This example illustrates the importance of high fidelity in device orientation relative to animal orientation [5], [6], [10].

There appeared to be lower performance of the KNN for the wombat. It is possible this had occurred because there did not appear to be a significant visual difference between the ‘walking’ and ‘running’ gaits other than speed in this species. Thus, it is possible that the patterns of locomotion during these two gaits would have produced similar patterns of acceleration data, which would have been difficult to discern in the KNN feature space. Accordingly, for species where discernible differences in locomotory gaits are not apparent, we advocate grouping of gaits into a single ‘locomotion’ behavioural mode in order to improve the performance of the KNN.

### KNN and the Movement Ecology ‘Toolbox’

One movement ecology paradigm aims to explain animal movement phenomena by integrating optimality, cognitive, random and biomechanical paradigms for animal movement into a single framework [11]. However, one of the factors impeding advance here pertains to the practical difficulties of recording animal movements and quantifying the underlying motivations [11]. It is not trivial to produce new methodologies to address this. By developing methods to identify behavioural modes in free living animals, Nathan [13] argued that it was possible to infer links between the biomechanical, behavioural and ecological processes that drive animal movement, something which is impossible to do by recording location alone. Thus the development of a ‘Toolbox’ of methods, by which information can be collected on behaviour, location and environmental factors, seems particularly germane.

By using the KNN method set out in the present study, it is possible to elucidate behaviour automatically from data derived from tri-axial accelerometers with greater ease than previously developed methods. Putting this information into a positional context through the use of GPS telemetry [47] or dead-reckoning methods [6], [7], [48] should provide further integration of the paradigms set out in Nathan [11]. Furthermore, the daily diary sensory suite proposed in Wilson *et al.* [10] also collects information on environmental conditions such as temperature and depth, as well as tri-axial acceleration (for use in KNN) and compass heading (for use in dead-reckoning), offering a means to study behaviour, location and environment with a single archival logger. Now that analysis methods such as that described in the present study offer an accessible means to link behaviour to animal position, this may be the start of a data rich era for movement ecology [11].

## Supporting Information

File S1 and Table S1 **A Detailed breakdown of Accuracy, Precision and Recall values for each species at each threshold value.**
(DOCX)
