# Supplementary material for: Love Thy Neighbour: Automatic Animal Behavioural Classification of Acceleration Data Using the K-Nearest Neighbour Algorithm
Source: PLoS One. 2014 Feb 21;9(2):e88609. doi: 10.1371/journal.pone.0088609 (PMC3931648; doi:10.1371/journal.pone.0088609)
Supplement: File S1 and Table S1 — A Detailed breakdown of Accuracy, Precision and Recall values for each species at each threshold value. (DOCX) [file pone.0088609.s001.docx]

Supplementary Information

Below is the R script used in order to conduct k – nearest neighbour analysis on tri-axial accelerometer data, and may be copied and pasted into R’s own Script Editor. The “train1.txt” file needs to contain the data derived from a ground truthing period of direct observation. Behavioural labels need to be assigned to the data in this file and then saved within a single column in a file called “classes1.txt”. These data labels act as the algorithms ‘definitions’ for the data, so it’s important that the order of the data labels in the “classes1.txt” file correspond with the order of data in the “train1.txt” file. The data that you would like to classify (derived from wild or unobserved individuals) should be placed in the “test1.txt” file, in a similar format to that of “train1.txt” i.e. same axis order, 3 columns. There is no need for these files to contain time stamps, but these can be saved and added later for descriptive statistics etc.

--------------------------------------------------------------------------------------------------------------------------------------

#This script runs K nearest neighbour analysis on files "train1.txt" and "test1.txt"

#Data files should be in .txt format, with each accelerometer axis in its own column, no spaces in between.

#Axis order (e.g. X,Y,Z etc..) is unimportant provided it is consistent between training and testing files

#The behavioural labels of the training set should be saved into a .txt file with a single column

#The label order should correspond to the accelerometer data in the training file

library(stats)

library(class)

#load up train and testing files

train1 = scan("train1.txt")

test1 = scan("test1.txt")

#convert inputs into matrix

train1 = matrix(train1, byrow = T, ncol=3)

test1 = matrix(test1, byrow = T, ncol=3)

#load the classes in the training data

cl1a = scan("classes1.txt")

#set k

kk = 21

#run knn

kn1 = knn(train1, test1, cl1a, k=kk, prob=TRUE)

prob = attributes(.Last.value)

clas1=factor(kn1)

#write results, this is the classification of the testing set in a sinlge column

filename = paste("results", kk, ".csv", sep="")

write.csv(clas1, filename)

#write probs to file, this is the proportion of k nearest datapoints that contributed to the winning class

fileprobs = paste("probs", kk, ".csv", sep="")

write.csv (prob$prob, fileprobs)

Supporting Information Legends

Table S1. A Detailed breakdown of Accuracy, Precision and Recall values for each species at each threshold value.

|  | Wombat | | | Kangaroo | | | Dingo | | | Cheetah | | | Human | | |
| --- | --- | --- | --- | --- | --- | --- | --- | --- | --- | --- | --- | --- | --- | --- | --- |
|  | Accuracy | Precision | Recall | Accuracy | Precision | Recall | Accuracy | Precision | Recall | Accuracy | Precision | Recall | Accuracy | Precision | Recall |
| 0.9 | 0.558 | 0.77226 | 0.59342 | 0.91 | 0.96498 | 0.90182 | 0.733 | 0.97265 | 0.6939 | 0.732 | 0.89704 | 0.74003 | 0.92663 | 0.89704 | 0.74003 |
| 0.8 | 0.601 | 0.769 | 0.67895 | 0.885 | 0.88814 | 0.95273 | 0.799 | 0.95051 | 0.79634 | 0.764 | 0.87517 | 0.8121 | 0.94171 | 0.87517 | 0.8121 |
| 0.7 | 0.661 | 0.77734 | 0.77632 | 0.83625 | 0.82083 | 0.97455 | 0.818 | 0.91974 | 0.85244 | 0.768 | 0.84713 | 0.85586 | 0.94422 | 0.84713 | 0.85586 |
| 0.6 | 0.718 | 0.77662 | 0.88289 | 0.73625 | 0.7263 | 0.98909 | 0.832 | 0.88263 | 0.91707 | 0.764 | 0.80914 | 0.9112 | 0.94472 | 0.80914 | 0.9112 |
| 0.5 | 0.757 | 0.76899 | 0.97237 | 0.69 | 0.68922 | 1 | 0.826 | 0.83437 | 0.98293 | 0.778 | 0.79119 | 0.9704 | 0.94724 | 0.79119 | 0.9704 |

|  | Cormorant | | | Camels | | | Badger | | |
| --- | --- | --- | --- | --- | --- | --- | --- | --- | --- |
|  | Accuracy | Precision | Recall | Accuracy | Precision | Recall | Accuracy | Precision | Recall |
| 0.9 | 0.67451 | 0.86772 | 0.67937 | 0.7505 | 0.89503 | 0.78563 | 0.7125 | 0.95297 | 0.40914 |
| 0.8 | 0.75715 | 0.84119 | 0.84258 | 0.7825 | 0.87634 | 0.85319 | 0.7095 | 0.84091 | 0.47184 |
| 0.7 | 0.7705 | 0.81567 | 0.90555 | 0.8 | 0.85864 | 0.90295 | 0.6515 | 0.64055 | 0.59086 |
| 0.6 | 0.76478 | 0.79042 | 0.94366 | 0.817 | 0.84219 | 0.95393 | 0.5565 | 0.5192 | 0.77577 |
| 0.5 | 0.7705 | 0.77432 | 0.98923 | 0.8165 | 0.81892 | 0.99447 | 0.477 | 0.47342 | 0.99362 |
